# Access of Migrant Youths in Sweden to Sexual and Reproductive Healthcare: A Cross-sectional Survey

**DOI:** 10.34172/ijhpm.2020.123

**Published:** 2020-07-26

**Authors:** Mazen Baroudi, Faustine Nkulu Kalengayi, Isabel Goicolea, Robert Jonzon, Miguel San Sebastian, Anna-Karin Hurtig

**Affiliations:** ^1^Department of Epidemiology and Global Health, Umeå University, Umeå, Sweden.; ^2^The Public Health Agency of Sweden, Solna, Sweden.

**Keywords:** Migrants, Youth, Access to Healthcare, Sexual Health, Reproductive Health, Sweden

## Abstract

**Background:** This study aims to assess migrant youths’ access to sexual and reproductive healthcare (SRHC) in Sweden, to examine the socioeconomic differences in their access, and to explore the reasons behind not seeking SRHC.

**Methods:** A cross-sectional survey was conducted for 1739 migrant youths 16 to 29 years-old during 2018. The survey was self-administered through: ordinary post, web survey and visits to schools and other venues. We measured access as a 4-stage process including: healthcare needs, perception of needs, utilisation of services and met needs.

**Results:** Migrant youths faced difficulties in accessing SRHC services. Around 30% of the participants needed SRHC last year, but only one-third of them fulfilled their needs. Men and women had the same need (27.4% of men [95% CI: 24.2, 30.7] vs. 32.7% of women [95% CI: 28.2, 37.1]), but men faced more difficulties in access. Those who did not categorise themselves as men or women (50.9% [95% CI: 34.0, 67.9]), born in South Asia (SA) (39% [95% CI: 31.7, 46.4]), were waiting for residence permit (45.1% [95% CI: 36.2, 54.0]) or experienced economic stress (34.5% [95% CI: 30.7, 38.3]) had a greater need and found more difficulties in access. The main difficulties were in the step between the perception of needs and utilisation of services. The most commonly reported reasons for refraining from seeking SRHC were the lack of knowledge about the Swedish health system and available SRHC services (23%), long waiting times (7.8%), language difficulties (7.4%) and unable to afford the costs (6.4%).

**Conclusion:** There is an urgent need to improve migrant youths’ access to SRHC in Sweden. Interventions could include: increasing migrant youths’ knowledge about their rights and the available SRHC services; improving the acceptability and cultural responsiveness of available services, especially youth clinics; and improving the quality of language assistance services.

## Background

**Key Messages**

**Implications for policy makers**

- Migrant youths in Sweden face challenges in the process of accessing sexual and reproductive healthcare (SRHC) services.
- The knowledge of migrant youths about available health services and how to access them should be increased through, for example, the introductory health examination.
- Another measure to improve migrant youths’ access could include increasing the services’ acceptability through improved cultural competence of youth clinics’ staff and ensuring good quality language assistance services.
- These measures should target all migrant youths, but more focus is needed on the following groups: men, non-binary, those born in South Asia, those waiting for a residence permit and those with low economic status.

**Implications for the public**

This study examines migrant youths’ use of sexual and reproductive healthcare (SRHC) in Sweden and provides information about the obstacles they face before and after they use the services. The study shows that migrant youths generally have limited ability to fulfil their needs of healthcare services and that some groups are more affected than others. These groups include men, those who do not identify themselves as men or women, those born in South Asia, those waiting for a residence permit and those with low economic status. The study concludes that the following measures are needed to fulfil migrant youths’ needs for SRHC: (1) Increasing migrant youths’ knowledge about the Swedish health system and available health services. (2) Improving the understanding of cultural differences among the Swedish healthcare staff. (3) Improving the quality of language assisting (interpreting) during healthcare visits.

Migrants usually bear an increased burden of sexual and reproductive ill health and tend to live in a vulnerable situation regarding sexual and reproductive rights. For example, migrants in high-income countries have worse maternal health outcomes, higher risk of HIV, and higher risk of sexual violence than native populations.^1-4^ They also receive worse healthcare compared to people born in the hosting countries.^5,6^

Migration to Sweden has steadily increased during the last decades, but its pattern has changed over time. While labour market migrants were the main group of migrants before the 1970s, refugees and asylum seekers have become the main group over the last years, and among them a significant number of unaccompanied minors. The number of new migrants in Sweden reached its peak in 2016, when around 163 000 migrants were registered, 35 000 of them unaccompanied minors. Currently, around 19% of young people 16-29 years-old in Sweden are foreign-born. The largest groups of migrant youths in Sweden are born in Syria, Afghanistan, Iraq, Somalia, Eritrea, Poland, Thailand, and Iran, which reflects the heterogeneity in ethnicity and religion of this population.^7,8^

Literature from Sweden and other high-income countries indicates that migrants face several barriers in their access to sexual and reproductive healthcare (SRHC). Several studies have shown that stigma, cultural and language differences, as well as lack of cultural competence, miscommunication and discrimination from healthcare providers, decrease migrants’ access to SRHC. Other obstacles also hinder their access to care: migrants’ socioeconomic status, health literacy, language proficiency, fear of deportation, mistrust and a lack of knowledge about the healthcare system and the available services.^9-13^

Insuring the highest attainable standard of sexual and reproductive health (SRH) is a fundamental right of all people. To reach this state of health, people’s right to access information and services should be respected and met, and the state should strive to provide SRHC on equal terms.^14-17^

The right of equal health, including SRH, and healthcare for all residents is a prioritised public responsibility in Sweden.^18,19^ SRH services in Sweden cover a wide range of services including SRH education, information and counselling. Additionally, services related to contraceptives, antenatal care, postnatal care and delivery, abortion, infertility, sexually transmitted infections and reproductive cancers are offered.^20^

Despite all efforts to provide equal SRHC, there is some evidence showing that migrants in Sweden have less access to healthcare than the native population. This situation is related not only to lack of legal entitlement to access most services for undocumented migrants and asylum seekers but also to poor economic status, discrimination, fear of deportation and the unfamiliarity of these rights among both healthcare users and providers.^21-23^ The literature reveals that migrants feel less respected, informed or able to engage in care.^24^ Studies have also reported how migrants in Sweden have less access to cervical and breast cancer screening than the general population.^25,26^ The inadequate access to SRHC among migrants may contribute to worse outcomes, for example, those related to maternal health.^3,6^

Age also plays an important role in SRH and access to SRHC. The prevalence of sexually transmitted diseases and abortion is higher among young people than in the older population.^27^ However, despite this higher need for services, young people generally have reduced access to SRHC. Consequently, the migrant youths not only have less access to SRHC compared to adults but also less access than their non-migrant peers.^28^

Factors such as social and gender expectations and norms differ among migrant youths, which can influence their sexual practices, SRH and access to SRHC.^27,29^ This heterogeneity of migrant youths may result in different barriers to access SRHC for various groups. For example, migrants with lower socioeconomic status face more difficulties in accessing healthcare. Furthermore, some migrant women might refrain from using contraceptives or avoid visiting health services if they are not attended by female health professionals because of cultural reasons.^30-33^

 However, there is limited information on access to SRHC for migrant youths and the potential barriers to access them among various subgroups of migrants with different socioeconomic characteristics. In this study, we aimed to (1) assess migrant youths’ access to SRHC in Sweden, (2) examine socioeconomic differences in their access, and (3) explore the reasons behind not seeking SRHC. In order to fulfil these objectives, access is conceptualised as the opportunity to use healthcare, as described in the following theoretical framework.

###  Theoretical Framework 

In this study, we used an adapted version of Levesque et al framework of patient-centred access to healthcare. According to this framework, access is defined as: “the possibility to identify healthcare needs, to seek healthcare services, to reach the healthcare resources, to obtain or use healthcare services, and to actually be offered services appropriate to the needs for care.”^34^ We used this framework because it highlights access as a process centred around patients/users and defines access as the opportunity for these patients/users to fulfil their needs of healthcare.

**Figure 1:**
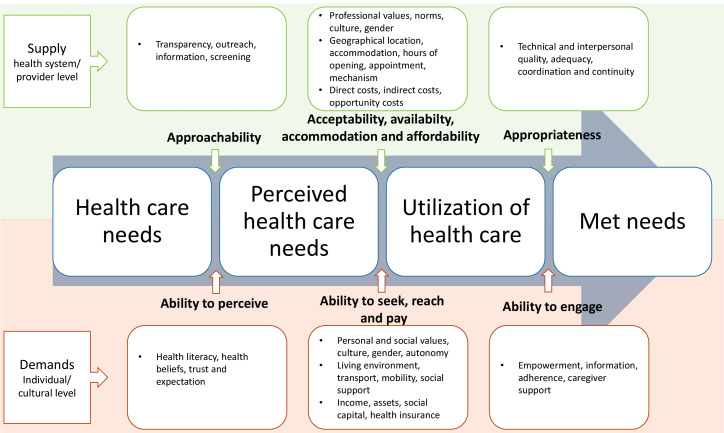
Theoretical Framework of Access to Healthcare. Adapted from Levesque et al framework of patient-centred access to healthcare.^34^

In our study, the access process consists of 4 stages: healthcare needs, perceived health needs, utilisation of healthcare, and healthcare consequences. The ability of healthcare users to move from one stage to another is related to individual, household and social factors (the demand side) and factors related to the healthcare institutions, organisations and providers (the supply side; Figure 1). The 4 stages can be defined as follows:

- *Healthcare needs:* the need to contact healthcare services regarding treatment, control or prevention of a health issue.
- *Perceived healthcare needs:* at this stage, people who have health needs realise their need for services. This stage is related to both demand factors such as health literacy, beliefs and trust as well as to the health system’s approachability, which is related to transparency, outreach, information and screening.
- *Utilisation of healthcare:* in our study, the stages of healthcare seeking, reaching and utilisation, which describe the actions taken after perceiving the need for services, were merged because of the difficulties in differentiating them in practice. This stage reflects people’s ability to obtain care after they realise their need for service, and it is related to:
  ♦ Personal autonomy and knowledge about one’s rights and healthcare options;
  ♦ The social norms and culture and if it is appropriate and acceptable for individuals to seek healthcare;
  ♦ The services’ availability and the ability to reach them on time including the availability of appropriate transport and the working hours flexibility of the users and healthcare professionals;
  ♦ The ability to pay for the services without catastrophic expenditure, including both the direct cost of treatment and the opportunity cost due to loss of income.
- *Healthcare consequences* (later called Met needs): the fulfilment of needs of services after utilisation, which is related to users’ ability to engage in treatment decisions and the appropriateness, adequacy and the quality of health services.

## Methods

###  Study Design

A cross-sectional survey was first designed based on previous national and international surveys; the Sexuality and Health among Young People in Sweden (UngKab15),^35^ Sexual and Reproductive Health and Rights in Sweden (SRHR17)^36^ and the British National Survey of Sexual Attitudes and Lifestyles (Natsal-3).^37^ The questions relative to the target group of migrant youths and the research questions were selected. The questionnaire was designed in Swedish and English and then forward translated into Arabic, Dari, Somali and Tigrinya, and tested in a pilot study to check language understanding and cultural appropriateness. After language and cultural adaptation, the surveys were backwards translated from Arabic, Dari, Somali, and Tigrinya into English and crosschecked with the original English questionnaire (the English version of the questionnaire is attached in Supplementary file 1).

###  Setting

The Swedish health system is mainly tax-funded, and all residents are entitled to social insurance that covers SRHC services. All SRHC services related to contraceptives, maternity and sexually transmitted infections that are of public health importance are provided for free to all people regardless of whether they have a residence permit. Visiting healthcare services is free of charge for all young people up to 18 years of age. Also, a free introductory health examination is supposed to be offered to asylum seekers to introduce them to the Swedish healthcare system, identify their needs and to “protect against the spread of infectious diseases” including HIV.^38^ Migrants also have the right to be assisted by an interpreter during health visits, free of charge.^38-40^ Migrants waiting for a residence permit decision and undocumented migrants are not entitled to other services free of charge, such as infertility treatments or consultations about relationships or sexual function.^41^

###  Study Population

The study targeted migrant youths. Migrant youths in this study refer to people 16 to 29 years old who were born outside Sweden, live in Sweden and have a residence permit or still waiting for a decision on their residence application. This age group is identified by the Public Health Agency of Sweden as priority group with regards to SRHR.^42^ We excluded second-generation migrants due to the low number of participants besides those born in the European Union, the United States, Canada, Australia and Japan since this group of migrants comes from countries with a similar context to Sweden.

###  Data Collection 

**Figure 2:**
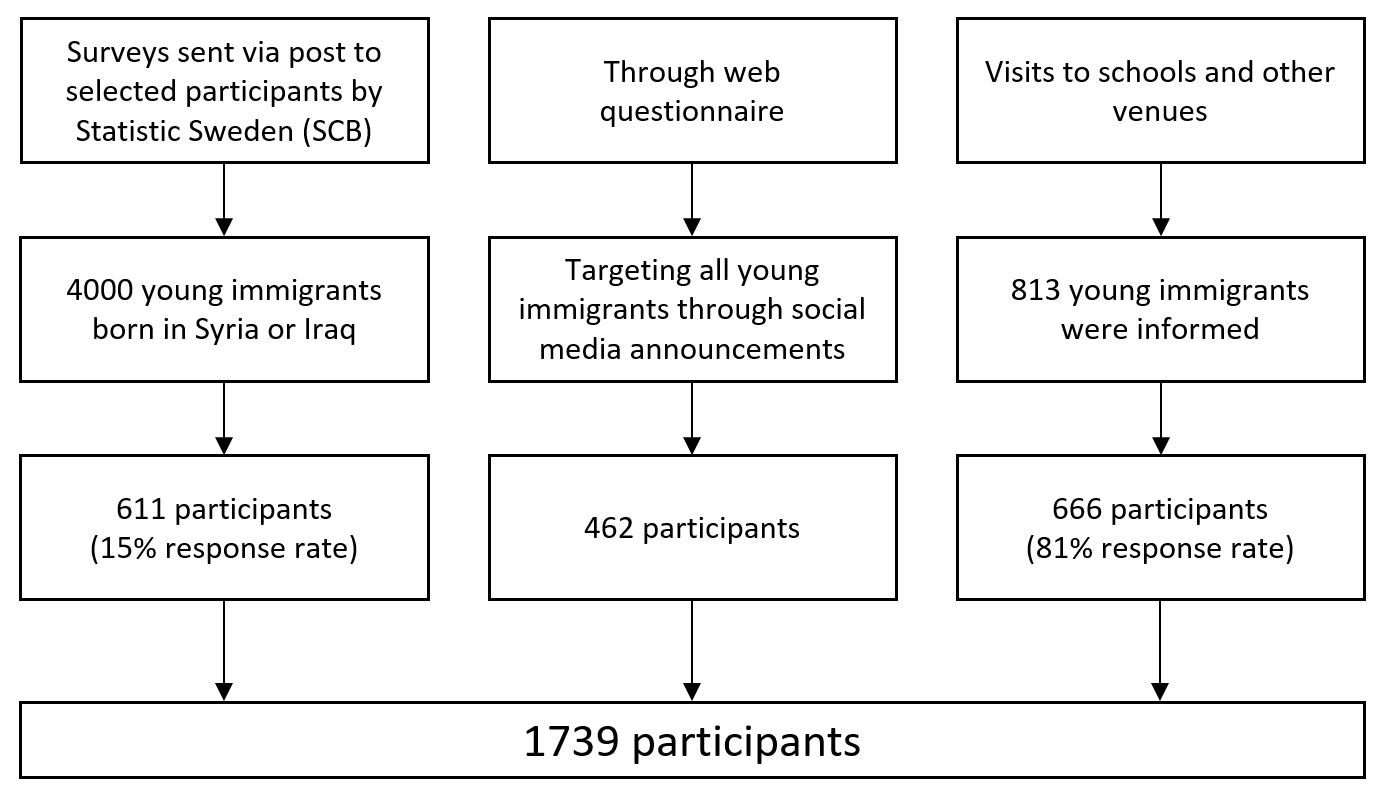
Study Participants and Data Collection Methods.

We used a mixed-mode design combining 3 recruitment strategies: (1) Surveys sent via post by Statistics Sweden (SCB) to migrant youths who moved to Sweden in the last ten years; (2) a web questionnaire was published on the university website with a targeted announcement via social media and; (3) a self-administered questionnaire through visits mainly to Swedish language schools and Swedish language introduction programs in secondary schools for new migrants (Figure 2). The survey was self-administered and was collected between March and September 2018. In total, 1739 migrant youths participated in this study (666 were met in person and 1073 through post or the web).

###  Measures

####  Operationalising the Framework 

It is necessary to measure the 4 stages of fulfilment of healthcare needs (hereafter called stages of access) to operationalise Levesque et al framework, ie, healthcare needs, perceived healthcare needs, utilisation and met needs. While measuring perceived needs and utilisation is quite straightforward, measuring healthcare needs and met needs is more problematic. As a proxy to measure healthcare needs, it is common to use health status, often measured by self-reported morbidity.^43^ However, the need for SRHC is not related only to morbidity, as a large proportion of the services provided are preventive and do not presuppose any disease.

Additionally, some services provided overlap with social services such as sexual abuse counselling.^44,45^ Therefore, Bradshaw’s taxonomy of social needs was used to estimate the need for SRHC.^46^ This approach includes various types of needs: the normative needs decided by an expert, the felt needs (those services wanted by the people) and the expressed needs when services are sought.^46^ Measuring met needs in our case is not as simple as the case of certain clinical conditions where consequences can be easily assessed by the number of cured cases or the number of complications. Therefore, in our study, met needs were estimated based on users’ satisfaction assuming that the users had fulfilled their needs of services.

####  Outcome Variables

**Figure 3:**
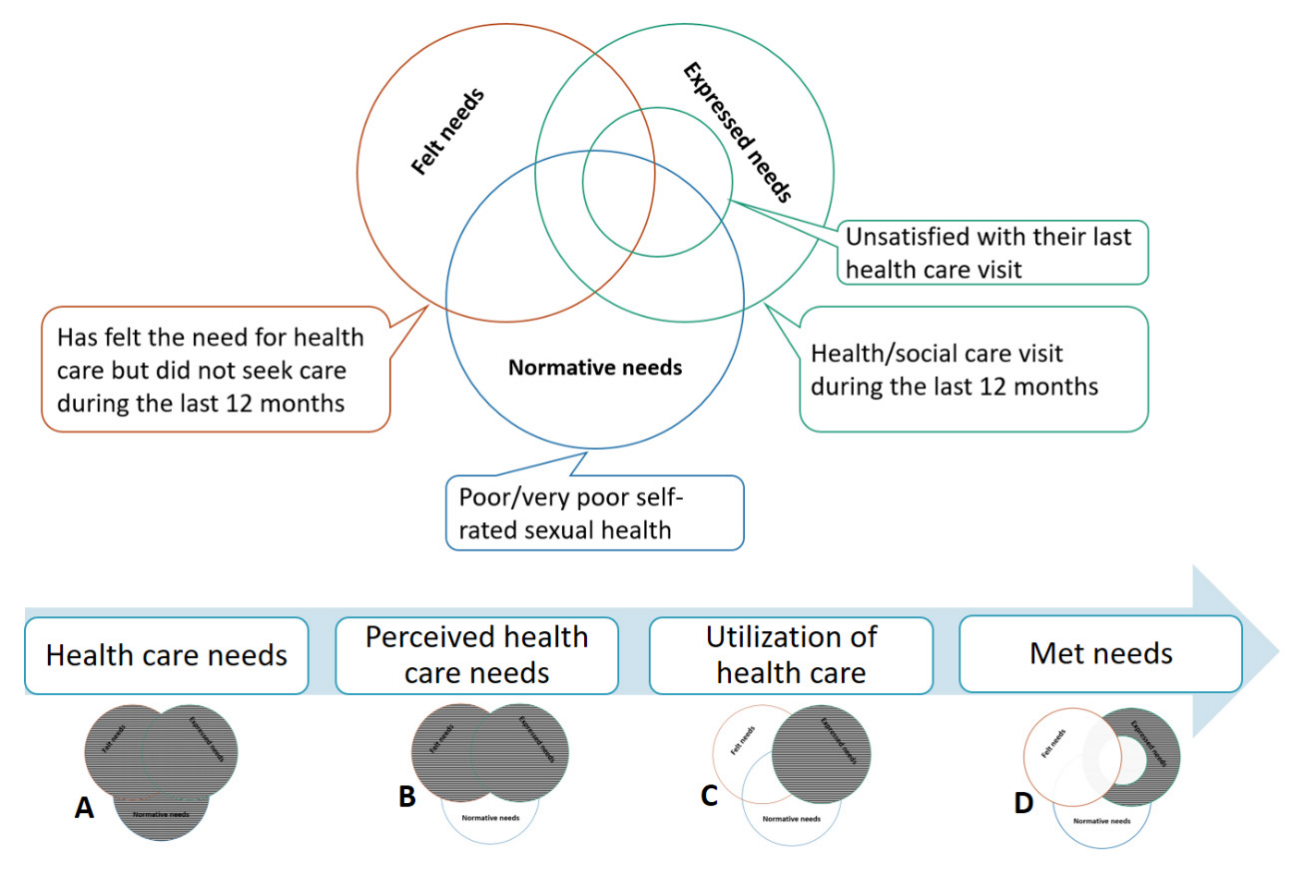
Definition of the 4 Stages of Access to SRHC Services. Note: Around 23% of the participants who visited SRHC had also reported that they had felt but did not express their needs (refrained from visiting SRHC). This category is reflected in the figure by the intersection between the two upper circles. Abbreviation: SRHC, sexual and reproductive healthcare.

 To measure the 4 stages of access, we defined that the participants had:

- *Need of services:* if during the last year, they had either visited the healthcare service (expressed needs), felt the need to seek healthcare but did not visit the healthcare service (felt but did not express their needs) or rated their sexual health as poor or very poor (normative needs; Figure 3, A).
- *Perceived needs:* if they expressed or felt the need for service in the last year, that is, they visited or refrained from visiting the service. To the contrary, participants who have poor or very poor self-rated sexual health and did not feel or express their needs were defined as having non-perceived needs (Figure 3, B).
- *Service utilisation:* if they visited an SRHC service in the last year (Figure 3, C).
- *Met needs:* if they had need of services, expressed their needs, were satisfied with the visit and did not refrain from seeking care. Participants were categorised as ‘satisfied’ if they were very satisfied, satisfied or neither satisfied nor dissatisfied during their last visit to an SRHC service. Those who were unsatisfied or very unsatisfied were categorised as ‘unsatisfied’ (Figure 3, D).

####  Covariates

 Those who visited an SRHC service were asked a follow-up question about what service they had visited, with options medical centre, youth clinic, gynaecological clinic and maternity clinic.

The reasons behind refraining from seeking healthcare included, among others, the following alternatives: 1) a *lack of knowledge,* ie, the participant did not know where to go; 2) *long waiting times* to get an appointment; 3) *language problems*, ie, the participant was not comfortable speaking Swedish; and 4) *financial reasons,* ie, the participant could not financially afford visiting a healthcare facility (see Supplementary file 1 for the full list of reasons explored).

*Gender* was classified as women, men, and ‘other’ including those who answered non-binary, do not know or do not want to answer. *Age* was grouped into 16 to 19 years, 20 to 25 years, and 26 to 29 years. *Region of birth* was categorised, according to the United Nations’ geographical regions, into the Middle East and North Africa (MENA), South Asia (SA), sub-Saharan Africa (SSA), and ‘other.’ *Education level* included 3 groups: 9 or fewer years of formal school education, ten to twelve years, and more than twelve years. *Economic stress* was used as a proxy for economic status. It was defined as the participant having difficulty in managing regular expenses for food, rent, bills, etc during the last twelve months. *Residence permit* was grouped into still waiting for a residence permit decision, had a residence permit year 2016 or later, or had a residence permit year 2015 or earlier. *Reason for migration* was classified into asylum seekers, including the United Nations’s quota refugees, family reunion, work, or other reasons of migration.

###  Statistical Analysis

To assess migrant youths’ access to SRHC (objective 1), we reported the descriptive analysis of the sample characteristics and the 4 stages of access. To examine the socioeconomic differences in access (objective 2), we estimated the adjusted prevalence of the 4 stages of access using margins post-estimation after a multivariate multiple linear regression controlled for gender, age, the region of birth, education level, economic stress, a residence permit and reason of migration.^47^ The adjusted prevalence and confidence intervals were reported considering a significant *P *value of less than 0.05. These estimates were then plotted to show the outcomes stratified by the socioeconomic characteristics. Finally, we explored the reasons behind refraining from seeking SRHC in a sub-sample of participants who reported the need for services through a descriptive analysis (objective 3). Stata 15 software was used for the analysis.

## Results

###  Sample Characteristics and Prevalence of the 4 Stages of Access

The sample consisted of 1739 migrant youths comprising around one-third of women (35%) and two-thirds men (63%). More than half of the participants were born in the MENA (57%) and around one-fifth in SA and SSA each. Around 26% of the participants had attended more than 12 years of formal education, whereas 45% had 9 years or less. Nearly half of the participants had experienced economic stress during the last year. Around 12% of the participants were waiting for a residence permit, and 56% had received a residence permit in 2016 or later. The most common reasons for migration were seeking asylum (72%) and family reunion (20%; Table 1).

**Table 1:** Sample Characteristics and Prevalence of the 4 Stages of Access by Individual Characteristics

	**n**	**(%)**	**Need of Services**	**Perceived Needs**	**Utilization**	**Met Needs**
			**%**	**%**	**%**	**%**
Total	1739	(100)	29.6	26.9	13.7	9.7
Gender						
Woman	586	(34.9)	30.3	28.8	21.6	15.9
Man	1060	(63.1)	28.1	24.9	9.7	6.4
Other	34	(2.0)	50.0	44.1	11.8	11.8
Age group (y)						
16 to 19	670	(37.8)	26.9	22.9	11.9	8.4
20 to 25	592	(33.4)	30.3	28.6	13.3	10.0
26 to 29	511	(28.8)	32.1	30.1	16.6	11.1
Region of birth						
MENA	989	(57.4)	27.5	26.1	13.7	9.6
SA	313	(18.2)	37.6	30.7	12.9	8.9
SSA	370	(21.5)	29.4	26.7	13.3	9.7
Other	51	(3.0)	18.8	18.8	14.6	12.5
Education level						
≤9 years	751	(44.9)	31.4	27.5	14.3	9.8
10 to 12 years	482	(28.8)	25.5	24.1	11.1	8.2
>12 years	441	(26.3)	32.0	30.6	15.5	10.7
Economic stress						
No	892	(52.4)	24.5	22.9	12.7	9.5
Yes	810	(47.6)	35.5	31.5	14.8	10.0
Residence permit						
Still waiting	188	(11.6)	42.3	36.3	12.1	7.1
2016 or later	911	(56.0)	27.3	24.9	14.6	10.0
2015 or earlier	528	(32.5)	27.5	26.3	13.2	10.8
Reason for migration						
Asylum	1108	(72.2)	31.5	28.6	13.3	9.3
Family reunion	313	(20.4)	25.3	23.6	16.7	13.4
Work	46	(3.0)	22.7	18.2	4.6	2.3
Other	67	(4.4)	35.8	31.3	13.4	9.0

Abbreviations: MENA, Middle East and North Africa; SA, South Asia; SSA, sub-Saharan Africa.

Around 30% of the participants had needs of SRHC. The majority of them perceived their need of services (26.9%); however, only 13.7% utilised the services, and only 9.7% met their needs (Table 1). The most commonly visited services were: (1) medical centres (8.3%); (2) gynaecological and maternity clinics (5.0%); and (3) youth clinics (4.5% of those 16 to 25 years old).

Men had an almost equal prevalence of need of services as women (30.3% and 28.1% respectively), while those who did not categorise themselves as either men or women had a higher prevalence of needs (50%). Compared to men (24.9%), women (28.8%) reported proportionally more perceived needs. Women (21.6%) also utilised the services twice as much as men (9.7%). Women (15.9%) achieved meeting their needs more than twice as often as men (6.4%). The younger the participants, the lower the prevalence in the 4 stages of access. Participants who were born in SA expressed a higher need of services and perceived needs but a lower utilisation and met needs compared to those born in other parts of the world. Compared to those who did not experience any economic stress, participants who had experienced economic stress had a higher prevalence of need of services and perceived needs but an almost equal prevalence of met needs. Participants who were still waiting for a decision regarding their residence permit had a higher prevalence of need of services and perceived needs but a lower prevalence of utilisation and met needs than those who had a residence permit (Table 1).

###  Differences in Access to Sexual and Reproductive Healthcare by Socioeconomic Characteristics

**Figure 4:**
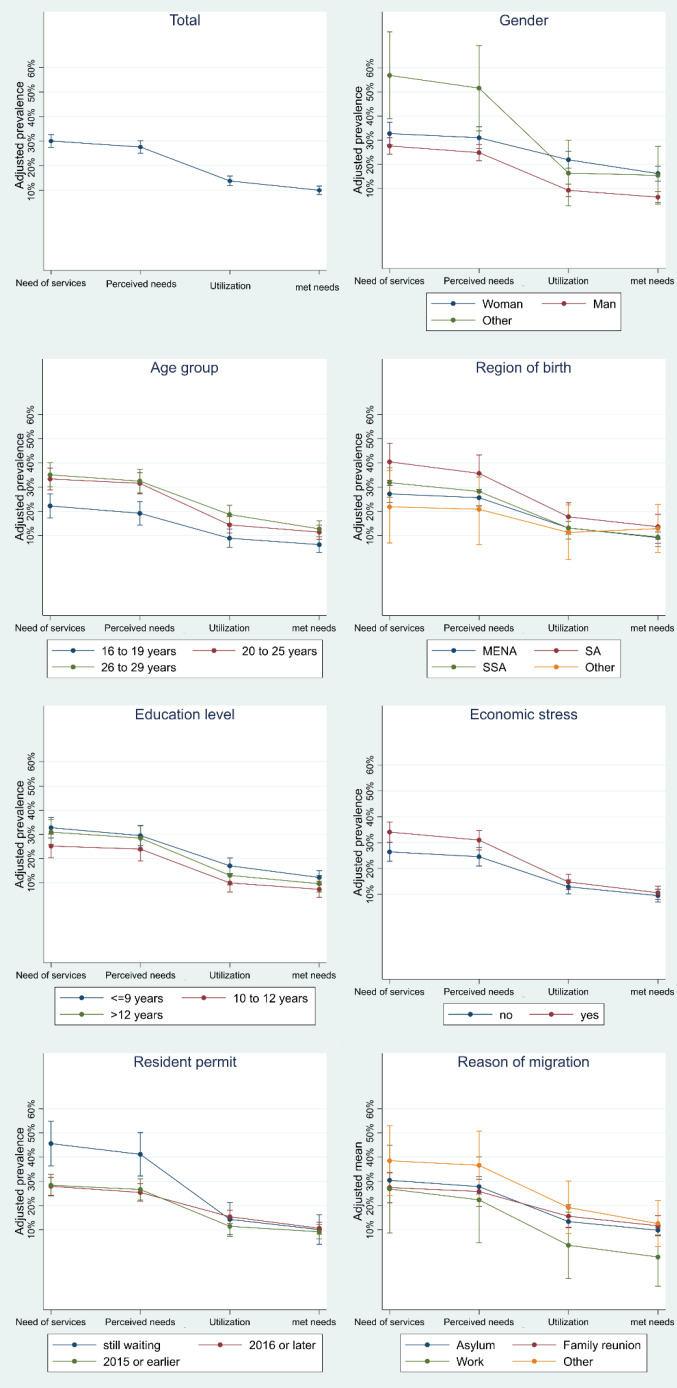
Comparison of Adjusted Prevalence of the 4 Stages of Access Among Migrant Youths by Socioeconomic Characteristics. Abbreviations: MENA, Middle East and North Africa; SA, South Asia; SSA, sub-Saharan Africa.

Figure 4 illustrates the process of access and the ability of the participants to move from one stage to another. It visualises the adjusted prevalence of the 4 stages of access stratified by the socioeconomic characteristics. The adjusted prevalence is provided in numbers in Table 2.

**Table 2:** Adjusted Prevalence and Their 95% CI of the 4 Stages of Access for Participants With Different Socioeconomic Characteristics

	**Need of Services**	**Perceived Needs**	**Utilization**	**Met Needs**
	**% (95% CI)**	**% (95% CI)**	**% (95% CI)**	**% (95% CI)**
Total	29.8 (27.3, 32.3)*	27.3 (24.8, 29.8)*	13.9 (12.0, 15.8)*	10.2 (8.5, 11.9)*
Gender				
Woman	32.7 (28.2, 37.1)*	30.7 (26.4, 35.1)*	22.2 (18.8, 25.5)*	16.3 (13.4, 19.3)*
Man	27.4 (24.2, 30.7)*	24.8 (21.6, 28.0)*	9.4 (6.9, 11.8)*	6.7 (4.5, 8.9)*
Other	50.9 (34.0, 67.9)*	44.0 (27.4, 60.6)*	10.4 (-2.5, 23.3)	10.9 (-0.5, 22.2)
Age group (y)				
16 to 19	21.6 (16.9, 26.3)*	18.4 (13.8, 23.0)*	8.7 (5.1, 12.3)*	6.4 (3.2, 9.5)*
20 to 25	32.3 (27.9, 36.6)*	30.6 (26.4, 34.9)*	14.4 (11.1, 17.7)*	11.3 (8.4, 14.2)*
26 to 29	36.4 (31.5, 41.2)*	33.8 (29.0, 38.5)*	19.4 (15.7, 23.1)*	13.3 (10.0, 16.5)*
Region of birth				
MENA	26.8 (23.4, 30.2)*	25.1 (21.7, 28.4)*	12.8 (10.2, 15.4)*	8.9 (6.6, 11.2)*
SA	39.0 (31.7, 46.4)*	35.0 (27.8, 42.2)*	18.3 (12.7, 23.8)*	14.2 (9.3, 19.1)*
SSA	32.5 (26.7, 38.3)*	28.8 (23.1, 34.5)*	14.1 (9.7, 18.5)*	10.1 (6.2, 14.0)*
Other	21.5 (7.1, 36.0)*	20.5 (6.4, 34.7)*	11.4 (0.4, 22.4)*	12.7 (3.1, 22.4)*
Education level				
≤9 years	32.2 (28.2, 36.3)*	28.8 (24.8, 32.8)*	16.5 (13.4, 19.6)*	11.9 (9.2, 14.6)*
10 to 12 years	25.2 (20.4, 30.0)*	24.0 (19.3, 28.7)*	10.4 (6.8, 14.1)*	7.7 (4.4, 10.9)*
>12 years	30.7 (25.6, 35.8)*	28.4 (23.4, 33.4)*	13.5 (9.7, 17.4)*	10.1 (6.7, 13.5)*
Economic stress				
No	25.7 (22.2, 29.2)*	23.9 (20.4, 27.3)*	12.6 (9.9, 15.2)*	9.4 (7.1, 11.8)*
Yes	34.5 (30.7, 38.3)*	31.3 (27.6, 35.0)*	15.4 (12.6, 18.3)*	11.0 (8.5, 13.6)*
Residence permit				
Still waiting	45.1 (36.2, 54.0)*	40.5 (31.8, 49.3)*	14.0 (7.2, 20.8)*	9.9 (3.9, 15.8)*
2016 or later	27.6 (24.1, 31.1)*	25.1 (21.6, 28.5)*	15.2 (12.5, 17.9)*	10.4 (8.1, 12.8)*
2015 or earlier	28.4 (24.0, 32.8)*	26.6 (22.3, 30.9)*	12.0 (8.7, 15.4)*	9.9 (7.0, 12.9)*
Reason for migration				
Asylum	30.4 (27.4, 33.4)*	27.8 (24.9, 30.7)*	13.7 (11.4, 16.0)*	10.1 (8.1, 12.1)*
Family reunion	27.1 (21.1, 33.1)*	25.2 (19.3, 31.1)*	15.4 (10.8, 20.0)*	11.8 (7.8, 15.8)*
Work	26.3 (9.7, 42.9)*	22.3 (6.0, 38.5)*	2.5 (-10.1, 15.1)	-1.4 (-12.5, 9.7)
Other	33.9 (20.4, 47.4)*	32.2 (19.0, 45.5)*	17.2 (6.9, 27.5)*	11.2 (2.1, 20.2)*

Abbreviations: MENA, Middle East and North Africa; SA, South Asia; SSA, sub-Saharan Africa. Statistical significance: * ≤.05.

 No statistically significant differences were found between men and women concerning the need of services (27.4% [95% CI: 24.2, 30.7] vs. 32.7% [95% CI: 28.2, 37.1] respectively) or perceived needs; however, men had a significantly lower prevalence of utilisation and met needs compared to women (met need; 6.7% [95% CI: 4.5, 8.9] vs. 16.3% [95% CI: 13.4, 19.3] respectively). Those who did not categorise themselves as men or women had significantly higher need of services (50.9% [95% CI: 34.0, 67.9]) compared to men. However, they did not differ statistically in the remaining 3 stages of access. Participants aged 16 to 19 years old had a significantly lower need of services and perceived needs than older participants, but they did not differ statistically in utilisation or met needs. Participants who were born in SA had significantly more need of services than those born in MENA (39% [95% CI: 31.7, 46.4] vs. 26.8% [95% CI: 23.4, 30.2]), whereas no statistically significant differences in the remaining 3 stages of access were found. Those who experienced economic stress had a significantly higher need of services and perceived needs but equal utilisation and met needs compared to those who did not experience any economic stress. A similar pattern was observed between those who were still waiting for a residence permit compared to those who had received their permit. There were no statistically significant differences in any of the 4 stages of access between participants with different education levels or with different reasons for migration.

###  Comparing the 4 Stages of Access

A comparison of the 4 stages of access among migrant groups with different socioeconomic characteristics showed a clear pattern of decreasing opportunities of accessing SRHC among all groups of migrants, which is illustrated by the downward slopes in Figure 4. Comparing between the estimated prevalence of need of services and perceived needs showed no statistically significant differences among any of the groups with different socioeconomic characteristics (in the total sample the prevalence of need of services was 29.8%, [95% CI: 27.3, 32.3] and the prevalence of perceived needs was 27.3% [95% CI: 24.8, 29.8]). In contrast, there were statistically significant differences between the estimated prevalence of perceived needs and utilisation for all socioeconomic groups except for migrants who were born in ‘other’ countries and those whose reasons for migration was a family reunion, work or ‘other.’ Furthermore, comparing the stage of utilisation with the met needs stage did not show statistically significant results in any of the socioeconomic groups (in the total sample the prevalence of utilisation was 13.9% [95% CI: 12.0, 15.8] and the prevalence of met need was 10.2% [95% CI: 8.5, 11.9]) (Figure 4, Table 2).

###  The Reasons Behind Refraining From Seeking Sexual and Reproductive Healthcare 

**Figure 5:**
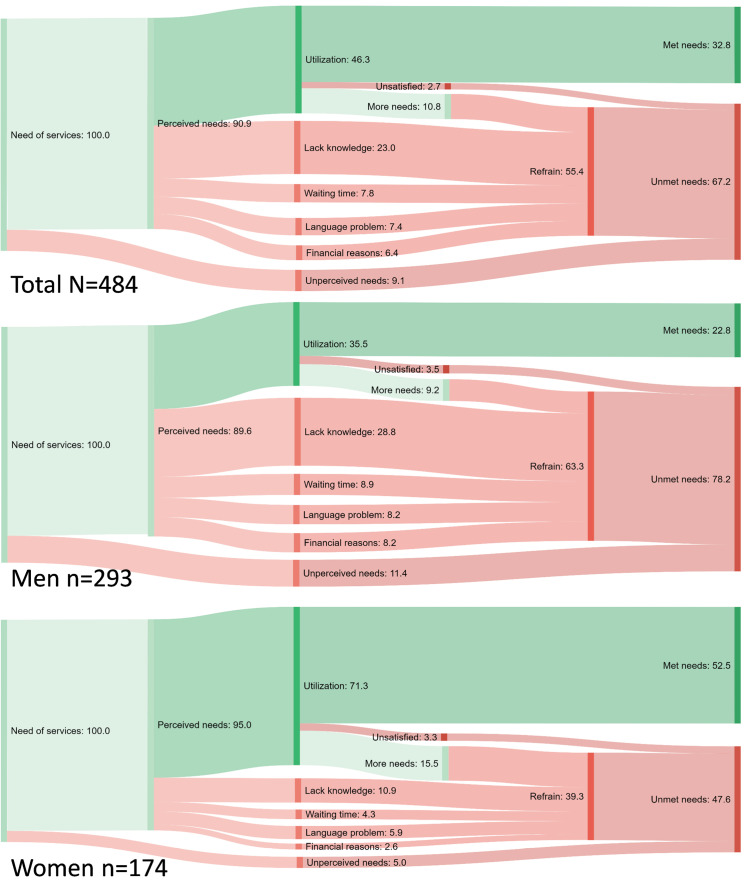
The 4 Stages of Access to SRHC and Reasons Behind not Seeking Care Among Participants Who Expressed the Need of Services Stratified by Gender.Abbreviation: SRHC, sexual and reproductive healthcare.

The analysis of the sub-sample of those who reported the need of services is shown in Figure 5 and Supplementary file 2. Figure 5 illustrates the 4 stages of access and the most commonly reported reasons for refraining from seeking SRHC services in the sub-sample of participants who reported the need of services stratified by gender (those who did not categorise themselves as men or women were not included due to low sample size). Of those who required services, 90.9% perceived their need (89.6% of men vs 95% of women), 46.3% utilised the services (35.5% of men vs 71.3% of women), and only 32.8% met their needs (22.8% of men and 52.5% of women). The lack of knowledge on how to navigate the Swedish health system was the most commonly reported reason for refraining from seeking SRHC (23%), and it was higher in men (28.8%) than in women (10.9%). Other reasons behind refraining were long waiting times (7.8%), language problems (7.4%) and financial reasons (6.4%).

## Discussion

 This study highlights the low ability of migrant youths in Sweden to access SRHC. Around 30% of the participants reported a need for SRHC during the last year, but just around half of them visited SRHC, and only one-third were able to fulfil their needs. While there was no difference in the need for SRHC between women and men, men faced more difficulties in accessing SRHC. Participants who did not categorise themselves as men or women were born in SA, experienced economic stress or were waiting for a residence permit expressed more need for SRHC and faced more difficulties in accessing SRHC. Reviewing the process of access indicated that migrant youths were able to perceive their needs of SRHC and were able to meet them once they utilised the services. However, their main challenge was in utilising the services after they had perceived the need. The most commonly reported reasons for refraining from SRHC were the lack of knowledge about the Swedish health system, long waiting times, difficulties in speaking Swedish and being unable to afford healthcare.

###  Low Ability to Fulfil Sexual and Reproductive Healthcare Needs

Our study showed that only 14% of all participants had utilised SRHC during the last year, and only 4.5% of youth had visited youth clinics. While a corresponding proportion of the general population in Sweden is not available, one study reported that 16% of the youths in northern Sweden had utilised youth clinics during the last 3 months.^28^ Other studies have also shown lower access to SRHC services among migrants compared to the general population in Sweden and other high-income countries.^4,25,26,48,49^

###  Reasons Behind the Low Access to Sexual and Reproductive Healthcare 

 Our study revealed that migrant youths were able to move from the need of services to perceived needs. However, the possible underestimation of the needs could have contributed to this result, as discussed later in the strengths and limitations section.

Although the study demonstrated no statistically significant results in the step between utilisation and met needs, the clear decreasing trend between the two stages suggests some barriers related to users’ ability to engage in treatment decisions and the appropriateness, adequacy and quality of health services. For example, our previous research illustrated that migrants visiting youth clinics in Sweden perceive the service as less equitable, having less quality, and they feel more disrespected by the staff and less supported by their parents, which could decrease their ability to meet their needs.^50^ The lower quality and adequacy of SRHC provided to migrants has also been reported in Sweden and other high-income countries.^4,48,51,52^

 Moving from perceived needs to utilisation exhibited difficulties for almost all socioeconomic groups. The participants who had refrained from seeking SRHC despite their needs identified some factors related to this move as their reasons behind their refraining. These factors included a lack of knowledge about the available service, difficulties in speaking Swedish, long waiting times and being unable to afford healthcare. These identified factors are in line with the barriers suggested in the Levesque et al framework in the steps between perceived needs and utilisation.

Our results agree with other studies that have shown that migrants usually have a lack of knowledge about the health system in the host country. This issue creates difficulties in navigating the new health system, which plays a role in the lower access of migrants to healthcare. For example, more than half the Thai women in Sweden did not know where to seek help related to SRHC, which lead to Thai women having 6 times less odd to utilise healthcare.^10,11,13^ While newly arrived migrants are often invited to an introductory health examination, this examination is often perceived by migrants as just a measure for HIV control, rather than an assessment for self-perceived health needs or an introduction to the Swedish health system.^53,54^

Language and cultural differences make it challenging to communicate effectively, resulting in lower acceptability and accessibility of healthcare.^11,53,55^ Migrants in Sweden have the right to be assisted by an interpreter during healthcare visits. However, a study reported that, in many cases, interpreters are either not utilised or lack professionalism and competency.^56^

###  Differences Between Socioeconomic Groups

Our study was able to identify various groups of migrant youths facing more difficulties accessing SRHC: men, those who did not categorise themselves as men or women, those who experienced economic stress, those born in SA or those waiting for a residence permit. The participants of the latter two groups consist mainly of young male asylum seekers from Afghanistan (most probably unaccompanied minors). The vulnerability and the inadequate care for this group was reported in previous research.^57,58^

The lower access to SRHC among men compared to women is in line with the literature from the general population in Europe.^59^ Male youth particularly underutilise youth clinics in Sweden as only around 10% of youth clinics visitors are men. The fact that most of the youth clinics’ staff in Sweden are women might make them less comfortable for young men.^60,61^ Another reason of lower access among men compared to women could be the lower level of knowledge about their services shown in this study. Reproductive health services targeting women, such as gynaecological and maternity clinics, could be more familiar to them since these services are probably available in their countries of origin. This familiarity with the services could contribute to women’s higher access to SRHC.

Previous research has shown disparities in prenatal care utilisation and maternal health outcomes between migrants of different ethnicities and those who were born in the host country.^62-64^ However, these ethnic disparities are often justified by the socioeconomic status among different groups of migrants.^33,65^ Though high economic status has been associated with more use of youth clinics in Sweden,^28^ our study has also shown differences between migrants born in different regions even after controlling for economic status. This observation highlights the vital role that the cultural background and experiences can play in accessing healthcare.

Studies have highlighted asylum seekers and undocumented migrants as particular groups underutilising healthcare both in Sweden and in other European countries.^21,66^ Our study supports these findings by showing less access to SRHC among those who were waiting for a residence permit in Sweden.

###  Strengths and Limitations

Access to healthcare is often conceptualised as the opportunity to use health services.^67-70^ However, most scholars operationalise this concept by measuring the utilisation of services rather than the opportunity to use them.^71-73^ Measuring utilisation can identify which group is facing inequalities in utilisation, but it is unable to suggest the reasons behind these inequalities; consequently, linking this approach with policies and practices is challenging.^69^ For example, identifying low utilisation of the family planning programme among one group does not explain how utilisation should be improved by policy practices.

 In contrast, the Levesque et al framework of access, which conceptualises access as a process, enabled us not only to pinpoint the groups that face more challenges in accessing healthcare but also revealed the stage of the access where the participants were facing more challenges. Therefore, we recommend measuring access as a process (the 4 stages of access) to make the studies more relevant to policymakers.

In measuring need of services, normative needs were estimated based on self-reported sexual health. This approach probably underestimated migrants’ needs of SRHC, as it did not include reproductive health status or other services such as the recommended screening for cervical cancer. Additionally, the validity and reliability of the questionnaire and the self-reported sexual health are yet to be determined. However, we can foresee that this measure can gain more acceptability and be used more often in the future, in the same way as the single question self-rated health.^74,75^ Furthermore, classifying those who answered “neither satisfied nor dissatisfied” with the services as having fulfilled their needs might overestimate the met needs.

 Even though migrant youths are hard to reach, the combination of 3 different methods of data collection enabled us to include various groups of participants (schooling and non-schooling). It contributed to expanding the diversity of participants. However, we cannot exclude that some youth could not be reached for different circumstances. The study included a relatively large sample size with participants, from 56 different countries living in Sweden, reading one of our questionnaire’s 6 language formats. However, some groups of migrants were not included in our sample if they were not comfortable reading one of these languages. Even though we asked the participant not to respond to the survey if they have already answered it on another mode of data collection, there is still the risk of some duplicate responses.

 Moreover, participants answering the survey through the web or via post were unable to ask for assistance, which might have led to the misunderstanding of some questions. For example, migrant youths might not have the knowledge about which services are included under SRHC; however, examples of SRHC services were written in the questionnaire and explained to the participants who were met personally. The questionnaires collected in schools or sent home could have been influenced by the lack of privacy due to the attendance of classmates or family members. However, when meeting the participants personally, all possible measures were used to insure privacy.

Due to data collection from language schools for new migrants, the low response rate of the home-sent questionnaires (15%) and the self-selected sample through the Internet, the selection and participation bias is a potential challenge in our sample. Our sample consisted of one-third women, and more than half the participants were born in MENA. However, we believe that our sample reflects the composition of migrants who have arrived in Sweden in the last 5 years. This group is important because they face more difficulties in accessing healthcare than other migrants; the literature indicates that the longer migrants stay in the host country, the better is their utilisation of health services.^76,77^

 The study used a cross-sectional design reviewing the last year of SRHC utilisation and making recall bias a potential problem. Another limitation of the cross-sectional design is the issue of temporality. For example, for those who answered that they had visited and also refrained from visiting SRHC, there is no possibility to know which act came first.

 During the analysis, migrant youths born in different countries were divided into groups based on the United Nations’ geographical regions. While this may not be optimal, the large number of countries represented made it challenging to analyse each country individually.

## Conclusion

 Our study shows a high need for SRHC services among migrant youths in Sweden with several challenges in the process of access. Certain groups were facing more challenges, especially those who were men or did not categorise themselves as men or women, were born in SA, were waiting for a residence permit or were experiencing economic stress. The reported reasons behind refraining from visiting SRHC services were the lack of knowledge about the Swedish health system, long waiting times, language barriers and financial concerns.

 Providing responsive SRHC for migrants is vital to fulfilling their rights to health. There is an urgent need to improve migrant youths’ access to SRHC in Sweden. To expand migrant youths’ access to SRHC, their knowledge about their rights, the Swedish health system and the available SRHC services should be increased using, for example, the introductory health examination. Other measures could include improving the services’ acceptability through strengthening the cultural responsiveness of available services and ensuring the quality of language assistance. These measures should target all immigrants with increased attention to the groups in situations of higher vulnerability.

## Acknowledgements

 The study made posible by the fund of the public health agency of Sweden (folkhälsomyndigheten). The authors are gratefull to all participants as well as to all contact persons in schools and other arenas and to all intrepreters and those who helped in translation and in data entry.

## Ethical issues

 The study was approved by the Research Ethics Committee at Umeå University [Dnr 2017/515-31]. Verbal or written informed consent was collected from all participants after they were informed about the study in an appropriate language. Participants’ autonomy, anonymity, confidentiality and rights of withdrawal were respected throughout the study.

## Competing interests

 Authors declare that they have no competing interests.

## Authors’ contributions

 Conception and design (MB, IG, MSS, AKH). Data obtaining and acquisition (AKH, FNK, MB). Analysis and interpretation of data (MB, FNK, IG, RJ, MSS, AKH). Drafting of the manuscript (MB). Critical revision of the manuscript for important intellectual content (AKH, MSS, IG, FNK, RJ). Statistical analysis (MB). Obtaining funding (AKH, IG, MSS, FNK, MB). Administrative, technical, or material support (MB, FNK, IG, RJ, MSS, AKH). Supervision (AKH, IG, MSS). Final approval of the submitted manuscript (MB, FNK, IG, RJ, MSS, AKH).

## Authors’ affiliations

^1^Department of Epidemiology and Global Health, Umeå University, Umeå, Sweden. ^2^The Public Health Agency of Sweden, Solna, Sweden.

## Supplementary files


Supplementary file 1. Migrants’ Sexual and Reproductive Health and Rights 2018 Questionnaire.

Supplementary file 2. Prevalence of the Stages of Access in the Sub-sample of Those Who Reported Need of Services.
