# Supplementary material for: Access of Migrant Youths in Sweden to Sexual and Reproductive Healthcare: A Cross-sectional Survey
Source: Int J Health Policy Manag. 2020 Jul 26;11(3):287–98. doi: 10.34172/ijhpm.2020.123 (PMC9278465; doi:10.34172/ijhpm.2020.123)
Supplement: Supplementary file 1 — Migrants’ Sexual and Reproductive Health and Rights 2018 Questionnaire. [file ijhpm-11-287-s001.pdf]

Supplementary file 1. Migrants' Sexual and Reproductive Health and Rights 2018 Questionnaire.

First there will be some questions about you and your background

1. I identify myself as:
   - ☐ Woman
   - ☐ Man
   - ☐ Non-binary
   - ☐ Other: Can you please specify? [ ]
   - ☐ Don't know
   - ☐ Don't want to answer.

2. How old are you?
   [|] years

3. In what country were you born?
   [ ]

4. How long have you been living in Sweden?
   [ ] year/s [ ] month/s

5. When did you obtain residence permit in Sweden?
   Year: [| | |]
   - ☐ I am still waiting for a decision

6. For what reason did you come to Sweden?
   - ☐ To apply for asylum
   - ☐ To live with my partner/ family who came earlier to apply for asylum here
   - ☐ To live with / marry a Swede
   - ☐ To live with / marry a non-Swedish living in Sweden
   - ☐ To live as a quota/resettled refugee selected by the UN's refugee agency
   - ☐ To work in Sweden
   - ☐ For another reason, what? [ ]

7. What is your religion?
   - ☐ Islam
   - ☐ Christianism
   - ☐ Another religion, specify: [ ]
   - ☐ Atheism
   - ☐ I don't want to answer

8. How many years of formal school have you completed?
   Including higher education if you attended a college or a university.

   [|] years
   - ☐ I have never attended a school

9. What is your present form of daily activity (occupation)?
More than one answer can be given.
- ☐ Work/ Employee
- ☐ Student/ Trainee
- ☐ Long term sick leave (more than 3 months)
- ☐ Job-seeker/unemployed
- ☐ Parental leave
- ☐ Other, please specify:

10. During the last 12 months, have you ever had difficulty in managing the regular expenses for food, rent, bills etc?
- ☐ No
- ☐ Yes, once
- ☐ Yes, more than once
- ☐ Yes, but before I moved to Sweden

11. With whom do you live?
ie, who do you live with for most of the week.
You can select several options.
- ☐ Nobody
- ☐ Family
- ☐ Spouse or partner
- ☐ Child/children (own children or other's children)
- ☐ Friend/s
- ☐ In a refugee accommodation (camp) / youth accommodation
- ☐ Other, please specify:

12. Do you need an interpreter when communicating with healthcare providers or other public services?
- ☐ Yes, always
- ☐ Yes, sometimes
- ☐ No, I can communicate in English
- ☐ No, a family member helps me
- ☐ No, I can speak Swedish

13. How much do you feel as part of the Swedish society?
- ☐ Fully
- ☐ To a great extent
- ☐ Somewhat but not completely
- ☐ Slightly
- ☐ Not at all

Now Some questions about your health, safety and social relationships

14. How would you rate your general state of health?
    - ☐ Very good
    - ☐ Good
    - ☐ Fair
    - ☐ Poor
    - ☐ Very poor

15. Do you have anyone you trust and can share your feelings with?
    - ☐ Yes
    - ☐ No

16. Can you get help from any person or persons if you are ill or have problems (eg, get advice, borrow things, help with shopping, repairs etc)?
    - ☐ Yes, always
    - ☐ Yes, most of the time
    - ☐ Not often
    - ☐ Never

17. In Sweden, do you ever avoid going out alone for fear of being assaulted, robbed or otherwise victimised?
    - ☐ No
    - ☐ Yes, sometimes
    - ☐ Yes, often

Now there will be questions on discrimination and physical violence

18. Have you ever been treated/addressed in a way that made you feel discriminated against or offended?
    You can select several options.
    - ☐ No → Go to question 22
    - ☐ Yes, in my country of origin
    - ☐ Yes, during my trip to Sweden
    - ☐ Yes, in Sweden

19. During the last 12 months in Sweden, have you been treated/addressed in a way that made you feel discriminated against or offended?
    - ☐ No → Go to question 22
    - ☐ Yes, once
    - ☐ Yes, several times

20. Was the discriminatory or offensive treatment associated with any of the following?
You can select several options.
- ☐ Ethnic origin or country of origin
- ☐ Sex (that you were born as a male or a female)
- ☐ Sexual orientation (whether you feel attracted to or prefer to have sex with people of same, opposite sex or both)
- ☐ Age
- ☐ Disability
- ☐ Religion
- ☐ Sexual identity (how you identify yourself as a man, woman…)
- ☐ Other, please specify:
- ☐ Don't know

21. Where were you discriminated against or offended?
You can select several options.
- ☐ At school
- ☐ At work
- ☐ At home by family or relatives
- ☐ In another residence or housing area
- ☐ In a public place (for example, bus, train, library or market)
- ☐ Through internet and social media
- ☐ In a healthcare facility
- ☐ In another public service (for example, Migrationsverket, Arbetsförmedling, Försäkringskassan or police station)
- ☐ In another place, please specify:

22. Have you, during the last 12 months been subjected to physical violence?
- ☐ No → Go to question 24
- ☐ Yes

23. Where did the violence happen?
You can select several options.
- ☐ At school
- ☐ At work
- ☐ At home by my partner/spouse
- ☐ At home by other relatives
- ☐ In another residence or housing area
- ☐ In a public place
- ☐ In another place, please specify:

Here are some questions related to sexual and reproductive health services
For example; services related to maternal and child health, family planning, pre- and postnatal care, sexually transmitted diseases, sexual violence, abortion, infertility.

24. Have you in the last 12 months visited healthcare service/social services regarding sexual or reproductive health issues?
- ☐ No → Go to question 29
- ☐ Yes
- ☐ Don't know

25. What was the main reason for your visit/s?
You can select several options.
- ☐ Information about contraception methods or to get contraceptives/condoms
- ☐ Information about sexuality and sexual health
- ☐ Sexual abuse or sexual violence
- ☐ To test for Sexually Transmitted diseases
- ☐ Abortion
- ☐ Antenatal care
- ☐ Delivery or postnatal care
- ☐ Other:

26. Which of the following clinic/s did you contact regarding sexual and reproductive health issues?
You can select several options.
- ☐ Medical centre
- ☐ Youth clinic
- ☐ Gynaecological clinic
- ☐ Midwifery clinic or maternity services
- ☐ Dermatology and Sexually Transmitted Diseases clinic (hud och std mottagning)
- ☐ Asylum/refugee health clinic
- ☐ Sex and relationship clinic (SESAM)
- ☐ The student health team (counsellor, psychologist or nurse)
- ☐ Health clinic in correctional treatment, state institutional care (SIS)
- ☐ Health clinic within social services/addiction care
- ☐ I don't know

27. During your last visit, how much do you agree with the following statements?

	Strongly agree	Agree	Disagree	Strongly disagree
a. I felt that I was treated with respect	☐	☐	☐	☐
b. The conversation or the examination happened in a way that no one else can see or hear us	☐	☐	☐	☐
c. The staff had an unprejudiced attitude towards me	☐	☐	☐	☐
d. I received treatment or help that met my expectations	☐	☐	☐	☐
e. I felt treated fairly without discrimination or offense	☐	☐	☐	☐

28. How satisfied were you with the service you received in your last visit?
- ☐ Very satisfied
- ☐ Satisfied
- ☐ Neither satisfied nor dissatisfied
- ☐ Somewhat dissatisfied
- ☐ Very dissatisfied

29. In the previous 12 months have you felt that you need sexual or reproductive healthcare but did not seek care?
- ☐ No → Go to question 31
- ☐ Yes

30. What was the reason/reasons you did not seek care?
More than one answer can be given.
- ☐ Did not know where to go
- ☐ The problem cleared up
- ☐ Waiting times too long
- ☐ Difficult to get through on the telephone
- ☐ Negative experience from previous visits
- ☐ I don’t trust the healthcare system in Sweden
- ☐ The clinic is very far from where I live
- ☐ I can’t financially afford visiting a healthcare facility
- ☐ I am not comfortable speaking Swedish
- ☐ Did not have time
- ☐ Other reason

31. Do you think that you need any of the following?
You can select several options.
- ☐ Opportunity to talk to someone about sexuality and relationships
- ☐ Free contraceptives
- ☐ Free condoms
- ☐ Clinics that are open during the weekends for testing and advice
- ☐ Information about where to get advice when the clinics are closed
- ☐ I don't need any of the above
- ☐ Other

Now there will be questions on sexuality and relationships

32. How would you rate your sexual health?
- ☐ Very good
- ☐ Good
- ☐ Fair
- ☐ Poor
- ☐ Very poor
- ☐ Don't know

33. How satisfied are you with your current sex life?
- ☐ Very satisfied
- ☐ Satisfied
- ☐ Neither satisfied nor dissatisfied
- ☐ Dissatisfied
- ☐ Very dissatisfied
- ☐ Not applicable

34. I feel sexually attracted..
- ☐ Only to men
- ☐ Only to women
- ☐ To men and to women
- ☐ Other, please specify: ________
- ☐ I don't feel sexually attracted to anyone at all
- ☐ Don't want to answer

35. To what extent are you, to others, open with your sexual identity/orientation?
- ☐ I am totally open → Go to question 37
- ☐ I am quite open
- ☐ I am partly open and partly not open
- ☐ I am not particularly open
- ☐ I am not open at all

36. Is your health affected by the fact that you are not living totally open with your sexual identity/orientation?
- ☐ Yes, my health is positively affected
- ☐ Yes, my health is negatively affected
- ☐ No, my health is not affected
- ☐ Don't know

37. Do you feel yourself limited by your family or your immediate surroundings in terms of whom you can have an intimate relationship with?
You can mark more than one alternative.
- ☐ No
- ☐ No, not by the others but I limit myself

- ☐ Yes, by my family/ parents/siblings
- ☐ Yes, by my country fellows
- ☐ Yes, because of my religious beliefs
- ☐ Yes, because of my sexual identity
- ☐ Yes, because of my sexual orientation
- ☐ Yes, because of a disability

- ☐ I miss a response alternative that suits me
- ☐ Don't know

38. Have you ever had sexual intercourse?
- ☐ Yes
- ☐ No → If you have never had sexual intercourse in your life you can go to question 49

39. How old were you when you had your first sexual intercourse?
If you are not sure, give an approximate age.
I was ☐☐ Years

40. How many persons did you have sex with during the last 12 months?
Give an estimate if you are not sure.
☐ Person/s

41. Which statement suits, generally, best for you?
- ☐ Me and my partner decide equally when to have sex
- ☐ I decide more often when to have sex
- ☐ My partner decides more often when to have sex
- ☐ None of the statements suits me

42. Do you feel that you can suggest to your partner how you want to have sex?
- ☐ Yes, always
- ☐ Yes, often
- ☐ Yes, sometimes
- ☐ No, rarely
- ☐ No, never

- ☐ Don't know

43. Do you feel that the sexual demands on you to perform are so high that it is a problem for you?
- ☐ No, never
- ☐ No, rarely
- ☐ Yes, sometimes
- ☐ Yes, often
- ☐ Yes, always

- ☐ Don't know

44. When you have sex with a new partner who wants to use a condom during sexual intercourse, how do you react then?
You can select several options.

☐ I think my partner does not trust me
☐ I think my partner has a sexually transmitted disease
☐ I feel disturbed as I don’t like to use a condom

☐ I don’t think it is necessary, because I trust this person
☐ I don’t think it is necessary, because we are using other contraceptive methods

☐ I think my partner is considerate/reasonable and I don’t have to worry afterwards
☐ I think it is good, as I would like to use condom myself

☐ I don’t know
☐ Not applicable

Now there will be some questions about the last time you had sex

45. If you think about the last time you had sex, do you agree with the following claims?

	No	Yes	Can't answer/ don't want to answer
a. I had sex in a way I wanted	☐	☐	☐
b. I had sex in a safe place where I felt secure	☐	☐	☐
c. I felt that I could suggest and use a condom or other contraceptive if I wanted to	☐	☐	☐

46. Did you/your partner used any of the following **to avoid pregnancy** during the most recent sexual encounter?
You can select several options.
☐ Yes, contraceptive pills or other hormonal method
☐ Yes, a condom
☐ Yes, intrauterine device
☐ Yes, a natural family planning (safe periods and/or withdrawal)
☐ Yes, emergency contraceptive pills

☐ No, but it was needed
☐ No, because we were planning to have a child
☐ No, I don't know how to do it or what to do
☐ No, because we had sex in a way which did not necessitate condoms or other contraceptives

☐ I'm unsure/don't know
☐ Other:

47. Did you/your partner **protect yourselves from sexually transmitted diseases** during the most recent sexual encounter?
You can select several options.
☐ Yes, a condom

☐ No, I don't think it is important
☐ No, but it was needed
☐ No, as we were planning to have a child
☐ No, I don't know how to do it or what to do
☐ No, as we had sex in a way that did not necessitate any protection

☐ I'm unsure/don't know
☐ Other:

48. Did you consume alcohol or use any kind of drugs, the last time you had sex?
☐ No
☐ Yes
☐ Don't want to answer
☐ Can't answer

Now there will be questions on sex against your will or paid sex

49. Have you ever experienced any of the following acts **against your will**?

		Yes	No, it did not happen	Don't know/ Not sure
a.	Harassments with sexual words or remarks.	☐	☐	☐
b.	Someone exposed him-/herself indecently in front of me	☐	☐	☐
c.	Someone touched my genitals or breasts	☐	☐	☐
d.	I had to masturbate for someone	☐	☐	☐
e.	I had vaginal sexual intercourse	☐	☐	☐
f.	I had oral sex	☐	☐	☐
g.	I had anal sexual intercourse	☐	☐	☐
h.	Sexually harassment through internet or social media	☐	☐	☐
i.	Someone distributed nude photographs of you on the Internet	☐	☐	☐
j.	Other:	☐	☐	☐

☐ No, I have never been subject to any of the above → Go to question 52

50. Was the person in the previous question:
You can select several options.
- ☐ Someone you have, or have been, in a relationship with (partner)
- ☐ A family member or a friend
- ☐ A teacher, boss, employer or a, supervisor
- ☐ A colleague or a classmate
- ☐ Someone I didn't know (a stranger)
- ☐ Other:

51. Did you talk or report to anyone about this?
You can select several options.
- ☐ Yes, I talked to a friend or a relative
- ☐ Yes, I talked to a teacher/school counsellor
- ☐ Yes, I informed my boss/employer
- ☐ Yes, I informed the social services (socialtjänst)
- ☐ Yes, I reported to the police
- ☐ Other, please specify
- ☐ No

52. Have you at some point…

		No	Yes	Don't know/ Not sure
a.	Received any compensation/payment for a sexual service?	☐	☐	☐
b.	Paid /given any compensation for a sexual service?	☐	☐	☐

Now there will be some questions about contraception and reproductive health

53. If you need contraceptives, do you know where to get them?
    - ☐ Yes
    - ☐ No
    - ☐ Not applicable

54. Have you or your partner ever been pregnant?
    - ☐ No → Go to question 58
    - ☐ Yes
    - ☐ Don't know

55. Have you or your partner had an abortion ie, intentional termination of a pregnancy?
    - ☐ No → Go to question 57
    - ☐ Yes, outside Sweden
    - ☐ Yes, in Sweden
    - ☐ I don't know

56. Who decided to have the abortion?
    - ☐ Me
    - ☐ My partner
    - ☐ Me and my partner together
    - ☐ Medical personal
    - ☐ Another person, please specify: ____________

57. Have you ever got a child, but you did not want to?
    - ☐ Yes
    - ☐ No

Here there will be some questions on sexually transmitted diseases, HIV and testing

58. How many sex partners have you had unprotected anal or vaginal intercourse with in the last 12 months?
    If you are unsure, please estimate
    ☐ Person/s
    ☐ I had no sex/unprotected sex during the last 12 months

59. Have you ever been tested or examined for any of the following diseases/s?
    you can select several options
    - ☐ Sexually transmitted diseases (for example Chlamydia, Syphilis or Genital herpes)
    - ☐ HIV
    - ☐ Hepatitis B or C
    - ☐ Yes, I was tested but I don't know for what
    - ☐ I don't know
    - ☐ No, I was never tested for any of the above → Go to question 64

60. Why did you test yourself?
you can select several options

- ☐ Got an invitation letter from a healthcare centre to undergo health screening as a new migrant in Sweden
- ☐ I started a new relationship
- ☐ I test myself at regular intervals
- ☐ I had unprotected sex with a casual partner
- ☐ I got notified as I had sexual contact with a person who got diagnosed with HIV or another sexually transmitted disease
- ☐ I was pregnant
- ☐ Other: ____________

61. Had anyone talked to you about the risks associated with unprotected sex and how to protect yourself, in the place where you last been tested?

- ☐ Yes, In Sweden
- ☐ Yes, in my country of origin
- ☐ No
- ☐ I don't know

62. Have you ever been diagnosed with any of the following disease/s?
you can select several options

- ☐ Sexually transmitted diseases (for example Chlamydia, Syphilis or Genital herpes)
- ☐ HIV
- ☐ Hepatitis B or C

- ☐ No, I was told that I was healthy
- ☐ Don't know, I did not receive the results after testing
- ☐ Don't know, I could not read or/and understand the results

} Go to question 65

63. Where was this disease discovered?

- ☐ In my country of origin
- ☐ During my travel to Sweden
- ☐ In Sweden

} → Go to question 65

64. Why **didn't** you test yourself?
You can select several options.

- ☐ I don't know where to get tested
- ☐ I don't want to know if I have a disease
- ☐ I was advised not to do so by a healthcare staff
- ☐ I was advised not to do so by a friend or a relative
- ☐ The opening hours of the clinic for testing don't suit me
- ☐ Don't believe that healthcare staff respect/conform to professional secrecy
- ☐ I'm scared of being recognised by the staff, by friends or country fellows
- ☐ I don't think it is needed
- ☐ Other: ____________

These questions are about your needs and sources of information for knowledge on sexual and reproductive health issues.

65. What are your main sources of information on relationships, sexuality, contraception and sexually transmitted diseases?
You can select several options.
- ☐ Websites produced by health and medical care such as, 1177, UMO.se and YOUMO.se
- ☐ Other websites on the Internet or social media
- ☐ Youth clinic
- ☐ A clinic within health and medical care (for example, sex and relationship clinic, health centre, midwife’s clinic, dermatology and STIs clinic etc)
- ☐ Education on relationships and sexuality in school
- ☐ Student health service (nurse, counsellor/curator)
- ☐ Friends
- ☐ Printed information material (Information sheets, newspapers)
- ☐ TV, radio
- ☐ Partner/s
- ☐ Father
- ☐ Mother
- ☐ Guardian (if somebody else besides mother and father)
- ☐ Siblings
- ☐ Other:

66. If you need more knowledge, do you know where to get this knowledge?
- ☐ Yes
- ☐ No

67. In which subjects do you think you need more knowledge?
You can select several options.
- ☐ The body and how it functions
- ☐ My rights and other people’s rights in Sweden in relation to sexuality
- ☐ The first sexual experience
- ☐ Sexuality and relationships
- ☐ How to negotiate using a condom with a partner
- ☐ How to avoid HIV and other sexually transmitted diseases
- ☐ How and where to test myself for HIV, chlamydia and other sexually transmitted diseases
- ☐ Family planning (How to; plan for a pregnancy, prevent unwanted pregnancy, use condom and contraceptives)
- ☐ I don’t need any more knowledge
- ☐ Other

68. In which language would you like to have this knowledge?
You can select several options.
- ☐ In my mother tongue
- ☐ In easy Swedish
- ☐ In English
- ☐ In other language, specify:

69. In what format or how would you like to receive information/knowledge?
You can select several options.
- ☐ Oral/verbal by a cultural mediator (someone who speaks my language and understands my culture)
- ☐ Oral/verbal by health care providers
- ☐ Written in form of pamphlets/leaflets
- ☐ Electronic messaging: Emails, Text message (SMS)
- ☐ Through a website on the internet
- ☐ Entertainment and media: TV, radio
- ☐ Visual: for example video, theatre

Here we have two final questions

70. How was it for you to answer this questionnaire?
You can select several options.
- ☐ The questions were important
- ☐ The questions evoked my emotions
- ☐ The questions were interesting to answer
- ☐ The questions were difficult to understand
- ☐ The questions were difficult to answer, they are too sensitive and private

71. If you have any further comments, please feel free to write them here:

THANK YOU FOR TAKING THE TIME TO COMPLETE THE SURVEY!
