# Supplementary material for: Access of Migrant Youths in Sweden to Sexual and Reproductive Healthcare: A Cross-sectional Survey
Source: Int J Health Policy Manag. 2020 Jul 26;11(3):287–98. doi: 10.34172/ijhpm.2020.123 (PMC9278465; doi:10.34172/ijhpm.2020.123)
Supplement: Supplementary file 2 — Prevalence of the Stages of Access in the Sub-sample of Those Who Reported Need of Services. [file ijhpm-11-287-s002.pdf]

Supplementary file 2. Prevalence of the Stages of Access in the Sub-sample of Those Who Reported Need of Services

	Need of services	Perceived needs	Utilization	Met needs
	n	%	%	%
**Total**	514	91	46	33
**Gender**				
Woman	174	95	71	52
Man	293	89	35	23
Other	17	88	24	24
**Age group**				
16 to 19 years	176	85	44	31
20 to 25 years	176	94	44	33
26 to 29 years	162	94	52	35
**Region of birth**				
Middle East & North Africa	270	95	50	35
South Asia	114	82	34	24
Sub-Saharan Africa	106	91	45	33
Other	9	100	78	66
**Education level**				
≤9 years	231	88	46	31
10 to 12 years	122	95	44	32
>12 years	140	96	48	33
**Economic stress**				
No	215	93	52	39
Yes	285	89	42	28
**Residence permit**				
still waiting	77	86	29	17
2016 or later	246	91	53	37
2015 or earlier	142	96	48	39
**Reason for migration**				
Asylum	344	91	42	30
Family reunion	77	93	66	53
Work	10	80	20	10
Other	24	87	37	25
